# Extending the spectrum of Ellis van Creveld syndrome: a large family with a mild mutation in the *EVC *gene

**DOI:** 10.1186/1471-2350-9-92

**Published:** 2008-10-23

**Authors:** Hakan Ulucan, Davut Gül, Julie C Sapp, John Cockerham, Jennifer J Johnston, Leslie G Biesecker

**Affiliations:** 1 Genetic Disease Research Branch, National Human Genome Research Institute, NIH, Bethesda, MD, USA; 2 Adnan Menderes University Medical Faculty, Department of Medical Genetics, Aydin, Turkey; 3 Gulhane Military Medical Academy, Department of Medical Genetics, Ankara, Turkey; 4 Children's National Medical Center, Department of Cardiology, Washington, DC, USA

## Abstract

**Background:**

Ellis-van Creveld (EvC) syndrome is characterized by short limbs, short ribs, postaxial polydactyly, dysplastic nails and teeth and is inherited in an autosomal recessive pattern. We report a family with complex septal cardiac defects, rhizomelic limb shortening, and polydactyly, without the typical lip, dental, and nail abnormalities of EvC. The phenotype was inherited in an autosomal recessive pattern, with one instance of pseudodominant inheritance.

**Methods:**

Because of the phenotypic overlap with EvC, microsatellite markers were used to test for linkage to the *EVC/EVC2 *locus. The results did not exclude linkage, so samples were sequenced for mutations.

**Results:**

We identified a c.1868T>C mutation in *EVC*, which predicts p.L623P, and was homozygous in affected individuals.

**Conclusion:**

We conclude that this *EVC *mutation is hypomorphic and that such mutations can cause a phenotype of cardiac and limb defects that is less severe than typical EvC. *EVC *mutation analysis should be considered in patients with cardiac and limb malformations, even if they do not manifest typical EvC syndrome.

## Background

Ellis-van Creveld syndrome (EvC, MIM 225500) is characterized by short limbs, short ribs, postaxial polydactyly and dysplastic nails and teeth [1]. The phenotype is variable and is inherited in an autosomal recessive pattern and parental consanguinity has been confirmed in about 30% of cases [2]. About two-thirds of affected individuals have a cardiovascular malformation, usually an atrial septal or atrioventricular septal defect [3]. The disorder was mapped to chromosome 4p16 and mutations in *EVC *and *EVC2 *genes, located in a head-to-head configuration, have been associated with this syndrome [3]. The EvC phenotype is variable but the range of variability has not been defined. We present here a clinical and molecular analysis of a large family with a phenotype that partially overlapped with EvC, but the affected individuals did not manifest many of the specific features of EvC.

## Methods

### Clinical report

#### Phenotype of Proband

**Figure 1:**
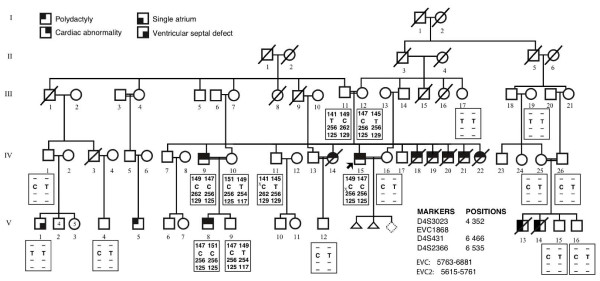
**Pedigree of the family.** The figure also shows haplotype analysis using markers encompassing both *EVC *and *EVC2*. The c.1868T>C variant is also presented in relation to the three markers used for linkage. The position of the markers is indicated in kilobase pairs from 4pter according to the March 2006 genome build. A hyphen (-) in the haplotype table means the marker was not typed in that person. The small "x" in the haplotype of individuals IV-11 and IV-15 indicates the suggested crossovers. The neonatal deaths of individuals IV-18 to IV-22 are thought to be caused by the same phenotype that affected the patients described in the case report. They had polydactyly and are thought to have congenital heart disease as they died in the neonatal period with cyanosis.

Individual IV-15 (Fig. 1) was examined at Gulhane Military Hospital, Ankara, Turkey. He presented at 24-years, was generally healthy, and had not undergone any major surgeries. Details of his birth history are not available. He is the product of a then 29-year-old healthy father and 21-year-old healthy mother who are thought to have a common ancestor, but the precise relationship is not known. He had four limb postaxial polydactyly with bilateral cutaneous syndactyly of toes 2–3. His toenails were dystrophic. Cubitus valgus, pectus deformity, narrow thorax, rhizomelic shortness of the limbs, mild scoliosis, and an S1 spina bifida occulta were also present. Cardiology consultation revealed ASD and VSD of the heart. He married his first cousin and they had two spontaneous abortions, but no information was available on the phenotype of these abortuses.

#### Detailed Phenotype of Individual IV-9

**Figure 2:**
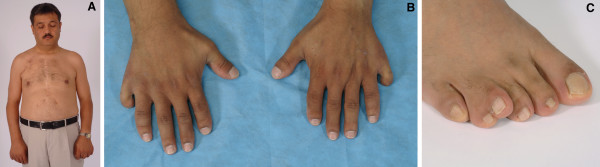
**Photographs of patient IV-9.** A) No facial or thoracic dysmorphic features were observed B) Postaxial polydactyly of the hands C) Postaxial polydactyly with partial cutaneous syndactyly of toes 4–5 of the right foot.

This 38-year-old male was examined at the NIH Clinical Center and Children's National Medical Center. He is the brother of the proband IV-15 (Fig. 2A). No information is available regarding the pregnancy or birth records. He was born with bilateral postaxial polydactyly of the hands (Fig. 2B) and unilateral postaxial (or central) polydactyly of the right foot (Fig. 2C), and he had poor exercise tolerance throughout childhood. At age 17 years he was diagnosed with an unknown type of septal defect and valvular abnormality. He underwent cardiac surgery at ages 17 and 30 years. The second operation was for replacement of the mitral valve and re-closing the septal defect. A pacemaker was placed after the first operation, but this was removed shortly thereafter as it was infected. The patient claimed that after removal, arrhythmia never recurred. He had a transient episode of aphasia or memory loss, associated with an incorrect adjustment of coagulation therapy and subsequently the symptoms resolved.

**Figure 3:**
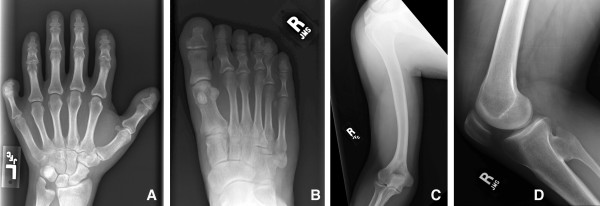
**Radiographs of patient IV-9.** A) Coned epiphysis of the left second middle phalanx and prominent styloid process of the ulna B) Irregular notched tip of the distal phalanx of the hallux C) Bowing of the right humerus D) Fusion of the right proximal tibia and fibula.

**Figure 4:**
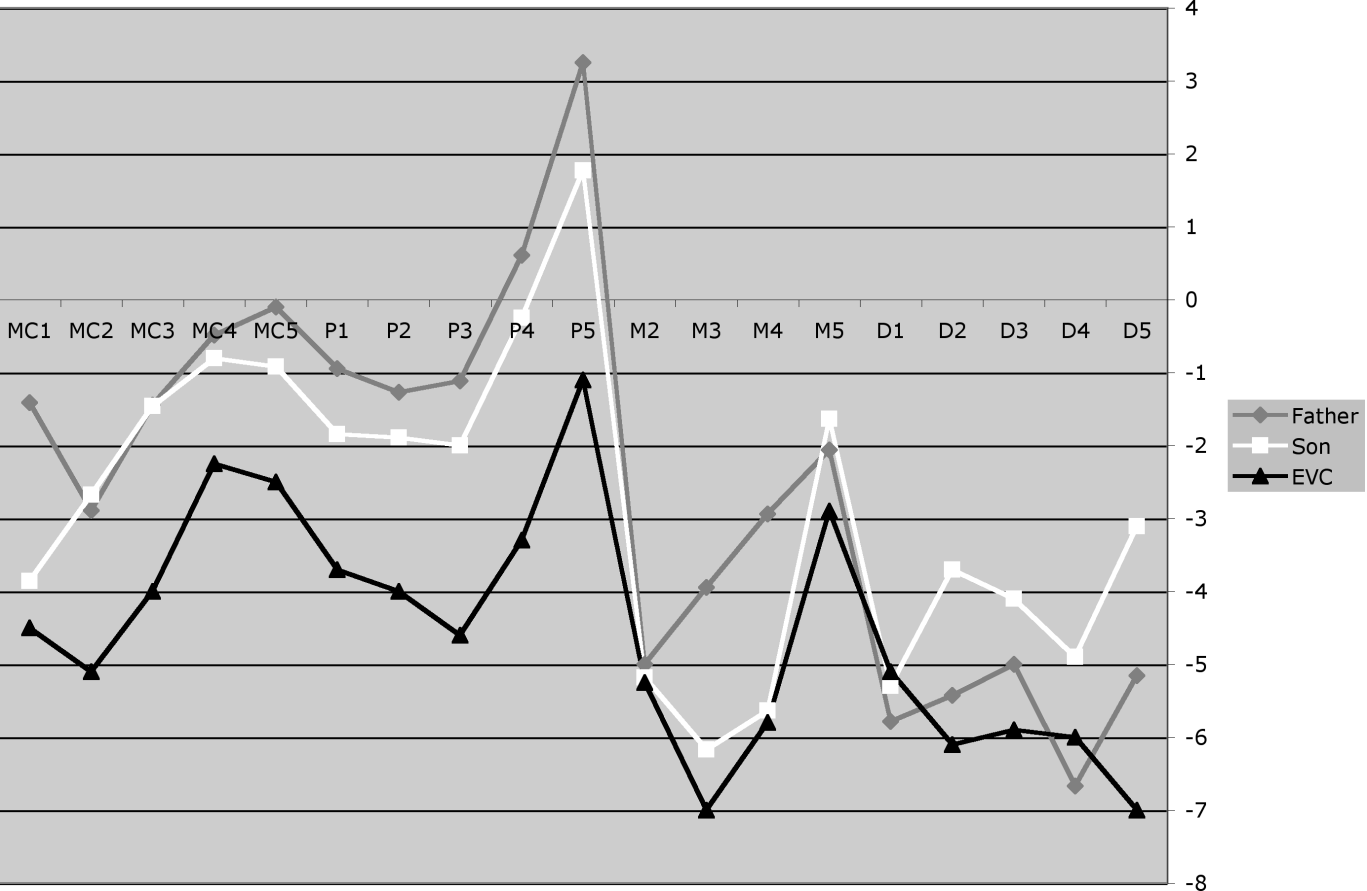
**Pattern profile analysis.** Profiles of the left hands of individuals IV-9 (Father) and V-8 (Son). This shows the individual profiles of the father and son and the published profile of patients with EvC [5].

He was examined at the NIH Clinical Center in 2007. His weight was 81.4 kg, height was 161.5 cm and head circumference was 57 cm (+ 0.8 SD) (Turkish norm) [4]. He had no facial dysmorphic features except for a relatively narrow and tall face and an occipital osteophyte. No extra frenulum or buccal adhesions were present. Hypodontia was evident including maxillary lateral incisors and mandibular first and second molars. No thoracic deformity was noted except scars due to previous operations. He had a bilateral intermediate type of postaxial polydactyly of the hands with a relatively small, but well formed, supernumerary triphalangeal digits. His dermatoglyphic pattern on the left was U,U,U,U,W and on the right was W,U,W,R,R (the sixth digits could not be assessed). He had short, but not hypoplastic, nails on the left hand on the second and third digits, on the right hand of the second digit only (Fig. 2B). Both sixth fingers had a small fingernail and they had normal passive mobility, but active flexion only occurred at the MCP joint. The second fingers of both hands were short with a stubbed or squared tip. His elbows had an increased carrying angle with normal mobility. He had postaxial polydactyly and partial cutaneous syndactyly of toes 4–5 of the right foot (Fig. 2C). He had laterally deviated and broad great toes with thick nails. The left foot was otherwise normal. Radiographs showed (in addition to the external findings above) a coned epiphysis of the left second middle phalanx and prominent styloid processes of the ulnae (Fig. 3A), an irregular notched tip of the distal phalanges of the halluces (Fig. 3B), bowed humeri (Fig. 3C), fusion of the right proximal tibia and fibula (Fig. 3D), and spina bifida occulta of S1. In addition, a pattern profile analysis of the left hand was performed (Fig. 4) [5]. Cardiovascular evaluation including echo- and electrocardiography showed mechanical mitral valve replacement and a small membranous VSD and first-degree trifascicular block. Renal ultrasonography showed a solitary 1.8 cm cyst in the right kidney. Mild splenomegaly was also noted. He had mildly elevated hemoglobin levels, suggestive of secondary polycythemia, possibly due to high altitude and obstructive sleep apnea. He had a normal 46,XY male karyotype (resolution between 300–400 bands). See Table 1 for a summary of phenotypic features in this patient and his son.

**Table 1:** Features of Ellis van Creveld syndrome, Weyer's acrofacial dysostosis and the family reported here

Feature	EvC	Weyer's	Patient 1 (IV-9)	Patient 2 (V-8)
Postaxial polydactyly	+++	++	+	+
Cardiac anomalies	++	+	+	+
Narrow chest	++	-	-	-
Short stature	+++	+	+	+
Distal limb shortening	++	-	-	-
Buccolabial fold, lip notch	+++	-	-	-
Short nails	++	++	+	+
Dysplastic nails	+++	+	-	-
Excess frenula	++	+	-	+
Hypodontia	++	+	+	+
Neonatal teeth	++	-	-	-
Small or bicuspid teeth	++	++	-	-
Genital anomalies	+			

In the "EvC" and "Weyers" columns, the symbol +++ indicates a nearly invariant finding, ++ indicates a frequent finding, + indicates an occasional finding, and – indicates a finding that is not considered part of that syndrome. In the columns describing the present patients + indicates a feature is present and – indicates a feature is absent.

#### Individual V-8

**Figure 5:**
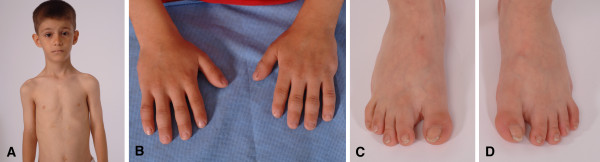
**Photographs of patient V-8.** A) Frontal view showing absence of dysmorphic features B) His bilateral extra digits were previously removed. Short digits and pseudoclubbing can be noted C) and D) Partial cutaneous syndactyly of toes 2–3 and clinodactyly of the second toes with broad toe tips.

**Figure 6:**
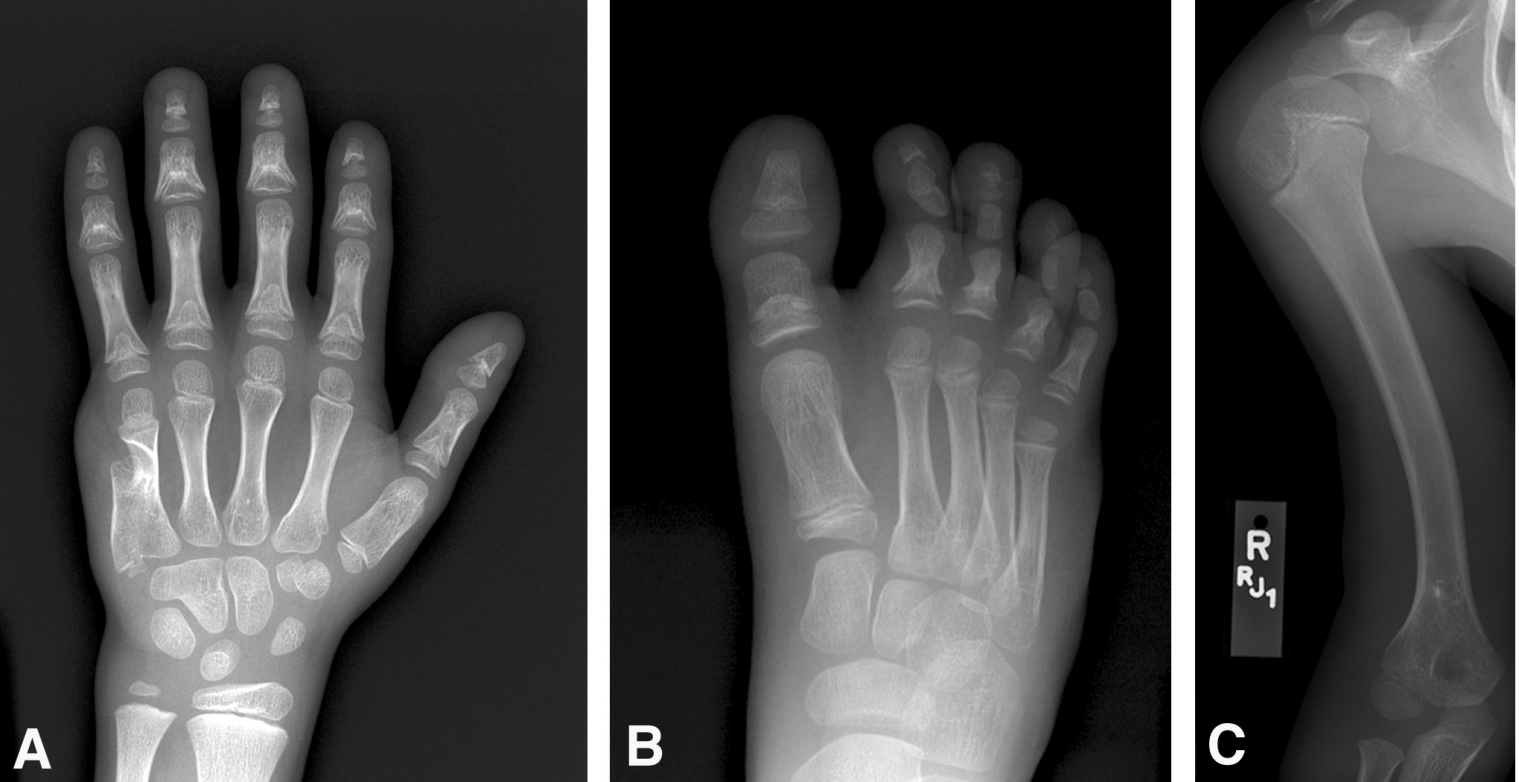
**Radiographs of patient V-8.** A) Residual appearance of postaxial polydactyly of the hand B) Abnormal appearance of some of the middle and distal phalanges of toes C) Mild lateral bowing of the humerus.

This 7-1/2-year-old male was also examined at the NIH Clinical Center and Children's National Medical Center. He, the son of IV-9, was the product of a gravida 2, para 2 23-year-old mother. The pregnancy was notable for abnormal fetal movements and poor weight gain. At the seventh month of gestation a fetal ultrasound examination revealed a protuberant anterior chest wall and short limbs, but polydactyly was not detected. After a spontaneous vaginal delivery his birth weight was 3,250 g (10–25^th^ centile), but his birth length and head circumference are unknown. Four limb polydactyly and a heart murmur were noted at birth. At the age of 6 months he was diagnosed with an atrial and ventricular septal defect and mitral valve insufficiency, which was repaired at age 4 years. At this time his polydactyly was repaired. Generally he was healthy. He is described as successful student in second grade attending regular school. His height was 113 cm, weight 17.4 kg (both 3–10^th ^centile) (Turkish norms [6]) and head circumference 49 cm. (Fig. 5A) Normative head circumference data for this population are disparate: 49 cm is <2^nd ^centile from one source [7] while this is well within the lower limits of normal, 3^rd ^– 10^th ^centile, from a second source [4]. Inner canthal distance was 2.5 cm (3^rd ^– 10^th ^centile), interpupillary distance was 5.1 cm (~10^th ^centile) and his outer canthal distance was 7.8 cm (~10^th ^centile) [4]. His nasal tip was broader than his nasal root. Oral examination revealed a single mandibular central incisor. He had two excess mandibular frenula, on each side of the mouth. He also had a central mandibular frenulum. The chest circumference was 53.5 cm (3^rd ^– 25^th ^centile) [8]. He had a mild lower pectus excavatum and several scars from cardiac surgery. He had a grade 5–6/6 holosystolic murmur. The upper extremities were notable for increased carrying angle, but with normal elbow mobility. Total hand length was 11.8 cm on the left, 12 cm on the right (both <3^rd ^centile) [8] (Fig. 5B). He had pseudoclubbing of the thumbs bilaterally. He had short, but not hypoplastic, fingernails 1 through 4 bilaterally with a normal fifth fingernail. His digits were proportionately short compared to his hands. Dermatoglyphic pattern on the left was U,U,U,U,W; on the right was U/W,U,U,U,A. He had lateral palm scars from postaxial digit removal. He had partial cutaneous syndactyly of the second and third toes bilaterally and clinodactyly of the second toes with broad toe tips (Figs. 5C,D). All biochemical and hormone blood and urine tests were within the normal range. Dental examination revealed absent maxillary and mandibular lateral incisors. Abdominal, renal and scrotal ultrasounds were normal. Radiographs showed, in addition to the external findings, residual appearance of post-axial polydactyly of hands and prominent cone-shaped epiphyses in the proximal and middle phalanges of all digits (Fig. 6A), atypical shapes of some of the middle and distal phalanges of the feet (Fig. 6B) mild lateral bowing of both humeri (Fig. 6C), spina bifida occulta of S1, and postoperative sternal wires. Pattern profile analysis of the hands was performed (Fig. 4). The bone age was about 6 years and 6 months. Echo- and electrocardiographic evaluation showed a residual cleft mitral valve and mitral regurgitation. He had a normal 46,XY karyotype (resolution 300–400 bands).

The affected family resides in a village of about 1,000 people in Eastern Turkey. Most of the inhabitants of the village claim ancestry from Central Asia and this particular extended family believe their ancestors migrated there from Kyrgyzstan some 150 years ago. The pedigree (Fig. 1) of the family shows that there are consanguineous marriages. The pedigree shows a number of neonatal deaths (e.g., children of III-11 and III-12), reportedly affected with polydactyly of the hands and feet. In addition to the surviving individuals described above, another patient (IV-14) survived to adult age after open-heart operations, but died at age 31 years of cardiac complications. Another nuclear family within the pedigree includes two patients, V-13 and V-14, with a single atrium and bilateral polydactyly of the upper and lower limbs, but without other features of EvC. They died at ages 3 months and 3 years, respectively, because of the heart condition. Another individual in another nuclear family (V-1) had a VSD without any other abnormalities.

### Subjects

The study included clinical analysis of the nuclear family described above (IV-9, IV-10, V-8, V-9) and molecular analysis of 15 additional family members (Fig. 1). The study was reviewed and approved by the IRB at NHGRI and the ethics committee of Ankara University Medical Faculty. Peripheral blood samples were collected in EDTA tubes and DNA was isolated by the salting out method (Qiagen, Inc.).

Because of the phenotypic overlap with EvC, candidate linkage analysis to the *EVC*/*EVC2 *locus was performed. The markers used in this analysis are shown in Fig. 1. Haplotyping was performed manually. The *EVC *and *EVC2 *genes were amplified from genomic DNA using standard methods, sequenced with the BigDye kit, and analyzed on an ABI 3100 sequencer (Applera Corp), with electropherogram analysis performed using the Sequencher program (GeneCodes, Ann Arbor).

## Results

Three markers were genotyped (Fig. 1) and all were partially or completely informative in the family. The haplotypes encompassing both *EVC *and *EVC2 *could not exclude linkage to this locus and therefore the genes were considered as candidates (Fig. 1).

### Changes in *EVC*

**Figure 7:**
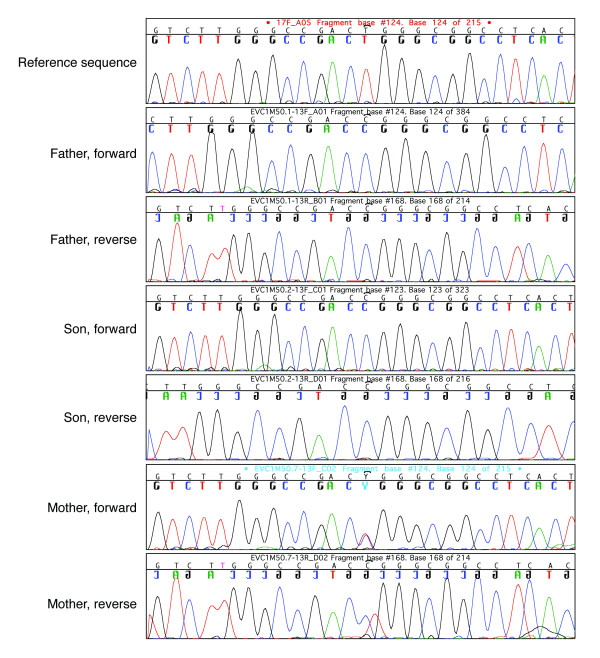
**Electropherograms of *EVC *exon 13.** Bidirectional sequence analysis shows c.1868T>C sequence variation, which is homozygous in the father (IV-9) and son (V-8), and heterozygous in the mother (IV-10).

**Figure 8:**
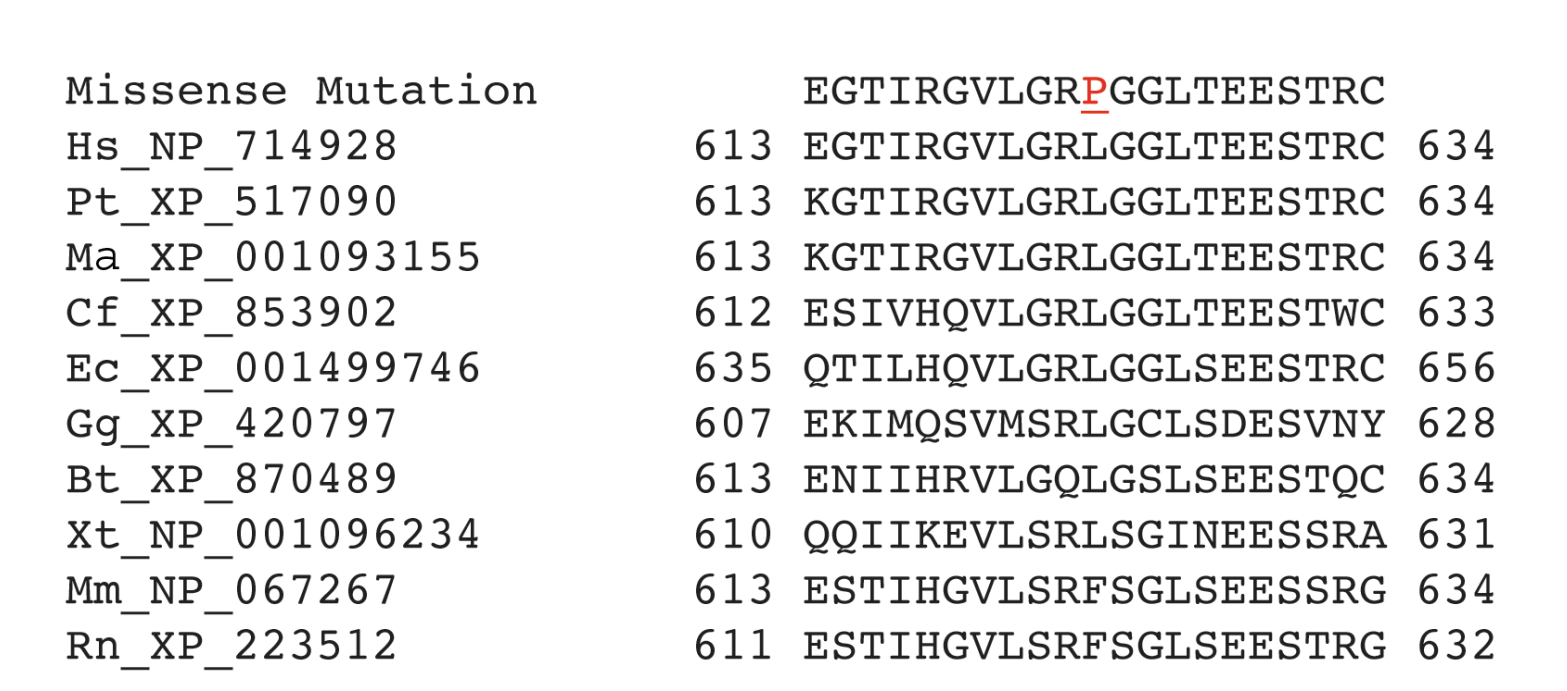
**Evolutionary sequence comparison.** Alignment of EVC sequence (first row) with nine orthologous protein sequences. The leucine at the position of change is conserved among seven orthologues. Hs is *Homo sapiens*, Pt is *Pan troglodytes*, Ma is *Macaca mulatta*, Cf is *Canis familiaris*, Ec is *Equus caballus*, Gg is *Gallus gallus*, Bt is *Bos taurus*, Xt is *Xenopus tropicalis*, Mm is *Mus musculus*, and Rn is *Rattus norvegicus*.

We identified seven sequence changes in the *EVC *when compared to the reference sequence. Six of the seven were recognized polymorphisms with appreciable minor allele frequencies (Table 2). We also detected a c.1868T>C sequence variation in Exon 13 of *EVC*, which predicts p.L623P that was homozygous in the father and son and heterozygous in the mother (Figs. 1 and 7 and Table 2). The remaining 16 samples were assayed for this variant and all obligate carriers were heterozygous and individual IV-15 was homozygous (Fig. 1). Individual V-1 was heterozygous for the variant but was previously judged not to be affected with the same phenotype as the rest of the family because he did not have polydactyly. This variant was not listed in dbSNP. This variant was not detected among 171 Caucasian control chromosomes. The *EVC *sequence was aligned with nine orthologous protein sequences (Fig. 8). The leucine at this position was conserved among seven orthologues. The other residues present at this location in the remaining two species were a phenylalanine in rat and mouse.

**Table 2:** *EVC *and *EVC2 *SNPs found to be informative in the nuclear family described in this manuscript

	SNP	Exon	Father (IV-9)	Son (V-8)	Mother (IV-10)
*EVC*	rs35870680	2	AA	AA	AG
	rs6446393	6	TT	TT	TT
	rs6414624	6	CC	CC	CC
	rs2302075	10	CC	CC	AC
	rs1383180	12	AA	AA	AG
	rs34870578	16	CC	CC	C-

*EVC2*	rs4689278	5	GG	GG	GG
	rs35103377	12	T-	T-	TT
	rs12511039	20	CT	CC	CT

The cells include the genotypes of each family member. The "-" symbol indicates a deleted nucleotide for an indel SNP. The third SNP in the *EVC2 *excludes the linkage centromeric of this marker.

### Changes in *EVC2*

Three sequence variants were detected in *EVC2 *(Table 2). Two heterozygous SNPs were detected in exons 14 and 20 of *EVC2 *in the father, whereas the son was homozygous. This excluded the telomeric portion of *EVC2 *from causing the phenotype, as both the father and son should be homozygous for the causative locus. All of these variants were recognized in dbSNP as variants with appreciable minor allele frequency. We conclude that none of these *EVC2 *variants are pathologic.

## Discussion

The phenotype of the affecteds shared some features with the description of EvC, but their features were not a good match for this disorder [1-3] (Table 1). The features in common with EvC include short stature, hypodontia, congenital heart defects, postaxial polydactyly of the feet and hands, and cone-shaped epiphyses of phalanges. However, these patients did not manifest many of the more distinct features of EvC. The patients reported here manifested rhizomelic shortening of the limbs rather than the mesomelic shortening that is typical for EvC. The pattern profile analysis is similar to, but less severe than, that seen in patients with EvC (Fig. 4). Also absent were the upper lip defects and hypertelorism. A major feature of EvC is a narrow thorax, and the patients reported here had only pectus excavatum with normal chest circumference. The nails were short, but were not hypoplastic or spoon shaped as reported for EvC. None of the characteristic genital abnormalities were observed in the two patients. The mucobuccal fold, which is the most striking and consistent oral manifestation of the disease was not present [2]. Neonatal teeth were also not reported in our patients. Overall, the patients had several of the less specific manifestations of EvC but few of the specific ones. For these reasons, we felt that they did not warrant a clinical diagnosis of EvC, but we felt that they shared enough features to warrant that mutations in *EVC *or *EVC2 *should be excluded.

The differential diagnosis includes McKusick Kaufman and Bardet Biedl syndromes. However, additional abnormalities of these two syndromes were not present in this family. Weyers acrodental dysostosis, an autosomal dominantly inherited allelic variant of EvC was excluded primarily because of the inheritance pattern, and again becuase the overall features in this family are not a good match for that disorder (Table 1). Distinctive radiographic features also distinguish the phenotype in this family from other chondrodystrophies such as achondroplasia, chondrodysplasia punctata, Morquio syndrome, short rib polydactyly, and cartilage-hair hypoplasia [9,10]. Sporadic cases with single atrium/atrioventricular canal malformation and hexodactyly may be variants of the syndrome [11,12].

The detection of haplotype inheritance in this family that was consistent with the recessive and pseudodominant inheritance observed in the pedigree led us to sequence *EVC *and *EVC2 *in their entirety. As these genes are separated by less than 20 kb in a head-to-head arrangement [13], few recombinants would be expected to occur between the genes, as was observed here. As mutations in either gene can cause EvC and there is no apparent genotype-phenotype correlation for this locus heterogeneity, we sequenced both genes. We detected a c.1868T>C sequence variation in Exon 13 of *EVC*, which predicts p.L623P. This variant was on the chromosome segregating with the phenotype in all tested family members (Figure 1). This variant is not described in dbSNP, was not present in a panel of 171 control chromosomes of Caucasian origin, and L623 is conserved across seven species, although this residue was a phenylalanine in two rodent species. This suggests that this residue is important, but not critical for the function of EVC. We conclude that *EVC *c.1868T>C, p.L623P is the causative mutation in this family and suggest that this allele is hypomorphic.

*EVC *has 21 coding exons spanning 120 KB of genomic DNA and encodes a 992 amino acid protein [1]. Only 25 mutations have been described in this gene (Table 3). Of those 25 mutations, 21 of them can be considered to be likely null mutations (frameshift, nonsense, splicing, and multiexon deletions). Of the remaining four mutations, one was a c.904_906delAAG, which predicts p.K302del. Three missense mutations have been described. These included p.S307P, p.R443Q, and p.Q896H. The p.S307P change has been associated with Weyer's acrodental dysostosis in a father of a girl with features of EvC. The daughter had, in addition to the p.S307P change, another frameshift mutation that led to EvC [1,14]. Another missense mutation, p.R443Q was heterozygous in a father and daughter, who both had no classical manifestations of EvC [1,11]. They both had postaxial polydactyly of the hands and feet, partial atrioventricular canal with common atrium, bilateral agenesis of the upper lateral incisors, enamel abnormalities, but normal stature. This phenotype is somewhat similar to the family presented here. It has been suggested that patients with single atrium or atrioventricular canal malformation and polydactyly may be variants of EvC. A homozygous mutation, p.Q896H was identified in a patient with classical EvC features [3]. Unfortunately no detailed information was given on the phenotype of this patient. Recently a 520-kb homozygous deletion comprising *EVC*, *EVC2*, *C4orf6*, and *STK32B*, caused by recombination between long interspersed nuclear element-1 (LINE-1) elements has been described in a consanguineous Egyptian family [15] (Table 3). The phenotype in that family is distinct from the family reported here because of mental retardation in addition to the classical features of EvC, which may be due to the involvement of *C4orf6 *and *STK32B*.

**Table 3:** The mutations described in *EVC*

cDNA change	Alternative or Protein prediction	Reference
c.174+1G>A	IVS1+1G>A	[3]
c.384+5_6GA>AC	IVS3+5_6GA>AC	[3]
c.703-1G>A	IVS5-1G>A	[3]
c.734delT	p.D246TfsX25	[1]
c.873_874insT	p.E292X	[3]
c.904_906delAAG	p.K302del	[1,3]
c.910_911insA	p.R304KfsX3	[1,3]
c.919T>C	p.S307P	[1,3]*
c.1018C>T	p.R340X	[1,3]
c.1328G>A	p.R443Q	[1]
c.1694delC	p.A565VfsX22	[3]
c.1777-2A>G	IVS12-2A>G	[3]
c.1813C>T	p.Q605X	[3]
c.1868T>C	p.L623P	
c.1886+5G>T	IVS13+5G>T	[1,3]
c.2089_2090insCA	p.R697TfsX14	[3]
c.2200C>T	p.Q734X	[3]
c.2278_2279insCGGC	p.R760PfsX7	[3]**
c.2304+2T>G	IVS15+2T>G	[3]
c.2456delG	p.M820WfsX107	[1]
c.2635C>T	p.Q879X	[1,3]
c.2688G>C	p.Q896H	[3]
c.1098+1G>A 5' splice-site mutation in intron 8		[15]
del EVC, EVC2, C4orf6, STK32B		[15]
del exons 10–21		[3]
del exons 12–21		[1]

*This designation was changed from that in the original report [3], wt is T, so the mutation nomenclature should be T>C. **This designation was changed from that in the original report [3] – the more 3' designation specified here is the proper nomenclature. These changes correspond to the specifications of the mutation nomenclature of the Human Genome Variation Society .

## Conclusion

We conclude that mutations in *EVC *can cause limb and cardiac abnormalities with other minor anomalies and that these phenotypes may not meet the threshold for typical EvC syndrome. We hypothesize that mutations in EVC can cause a broad range of clinical phenotypes and that mutations in this gene should be sought in patients who present with the dyad of septal anomalies and polydactyly.

## Abbreviations

EvC: Ellis van Creveld syndrome.

## Competing interests

The authors declare that they have no competing interests.

## Authors' contributions

Study design: LGB, JJJ, and HU. Clinical evaluation of patients at NIH and Children's National Medical Center: LGB, JCS, JC, HU. Clinical evaluation and sample collection of patients in Turkey: DG and HU. Generation and analysis of molecular data: LGB, JJJ, and HU. Preparation of the manuscript: LGB and HU. Administrative and funding support: LGB (NIH) and DG and HU (Gulhane Military Academy, Ankara and Adnan Menderes University).

## Pre-publication history

The pre-publication history for this paper can be accessed here:


